# Anthelmintic efficacy of fenbendazole and levamisole in native fowl in northern Iran

**DOI:** 10.1186/s13071-021-04605-9

**Published:** 2021-02-08

**Authors:** Atefe Saemi Soudkolaei, Gholam Ali Kalidari, Hassan Borji

**Affiliations:** 1 Department of Pathobiology, Faculty of Veterinary Medicine, Ferdowsi University of Mashhad, P.O. Box, 91775-1793 Mashhad, Iran; 2 Department of Clinical Sciences, Faculty of Veterinary Medicine, Ferdowsi University of Mashhad, Mashhad, Iran

**Keywords:** Domestic chicken, Fenbendazole, Levamisole, Anthelmintic efficacy, Iran

## Abstract

**Background:**

With the increasing number of free-range domestic chickens, helminth parasites have potentially become more of a threat to commercial flocks in recent years, and routine poultry deworming is needed to improve the efficiency of chicken production. The present study deals with a field trial to study the efficacy of two generally used anthelmintics, fenbendazole and levamisole, against gastrointestinal nematodes of domestic chickens in northern Iran.

**Methods:**

Of 45 domestic chicken flocks involved in the study, 20 flocks were selected to participate in fecal egg count reduction testing based on flock size from April 2017 to September 2018. The infected chickens were randomly divided into three equal groups of 30 each. Ninety chickens in the infected groups received one of the following treatments (d 0 of treatment): Group 1: 5 mg kg^−1^ body weight (BW) fenbendazole for 3 consecutive days; Group 2: 16 mg kg^−1^ BW levamisole; Group 3 control: placebo, water + DMSO (dimethylsulfoxide). The efficacy of the treatments was evaluated by comparing fecal egg counts in the treated and control groups.

**Results:**

Examination of three flocks of chickens from the control group showed that 95.0% of the animals were infected with gastrointestinal nematodes with an average geometric value of 361 eggs per gram of feces. Fenbendazole at a dose of 5 mg kg^−1^ BW for 3 days showed an efficacy of 83.7% (*P* ≥ 0.05), and levamisole at a dose of 16 mg kg^−1^ BW showed 71.8% efficacy (*P* ≥ 0.05) with geometric mean eggs in a gram of feces of 100 and 199.6, respectively. In general, fenbendazole and levamisole treatment led to significantly lower activity. The result of this study revealed that fenbendazole was a better and more effective dewormer than levamisole on the three Iranian domestic chicken flocks, but the difference was not significant. *Capillaria* spp. were the most generally resistant nematodes followed by *Trichostrongylus* spp. and *Amidostomum anseris*.

**Conclusion:**

Our results indicated that fenbendazole and levamisole effectively reduced the number of nemathodes in three Iranian domestic chicken flocks. Given the results of our study, resistance can be expected in the parasitic helminths of poultry. Additional larger scale studies are required to determine the prevalence of anthelmintic resistance in the poultry industry.
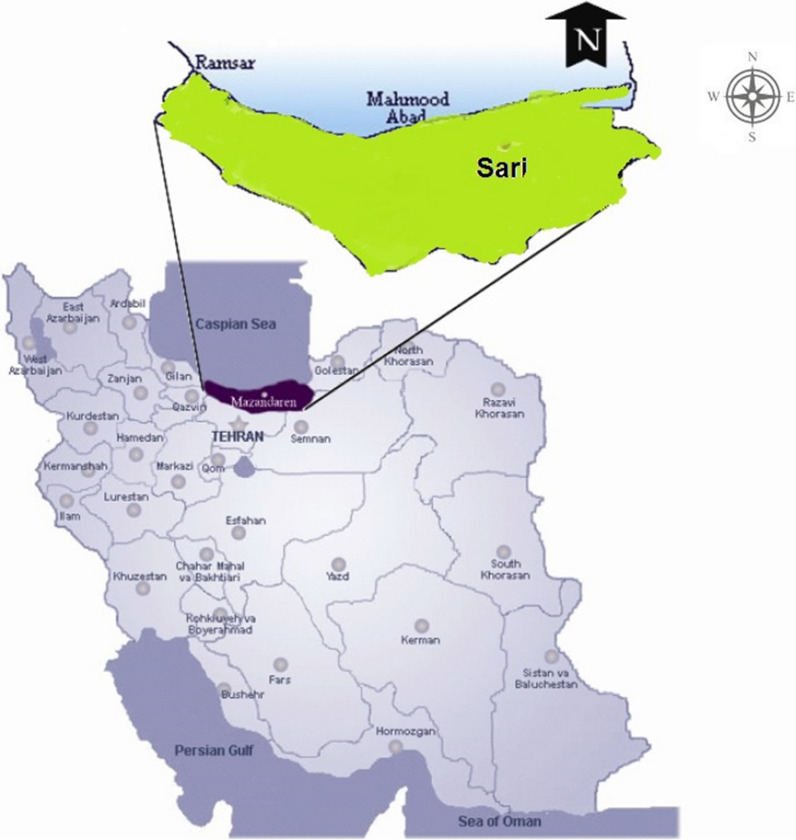

## Introduction

Domestic chickens play an important role in the economics of rural regions of small societies worldwide. Traditional free- range chickens provide a critical source of food and income for these people. However, the growing demand for chicken products from free-range production systems is hampered by the heavy burden of reemergence of diverse poultry helminths [1]. Helminth parasitism remains one of the most significant widespread infestations in rural chickens worldwide due to scavenging habits in free-range raising chickens, causing clinical and subclinical diseases, which hinders food production and chicken health. Consequently, practical parasite control approaches are required to support poultry health and production and hence to confirm the sustainability of poultry products. Not surprisingly, the control of gastrointestinal parasites in poultry is restricted by the high cost of anthelmintics, unreliable availability, and the growing occurrence of drug resistance.

In Iran, three broad-spectrum anthelmintic classes with different modes of action are listed for practice in livestock: the imidazothiazoles (e.g. levamisole), macrocyclic lactones (e.g. ivermectin), and benzimidazoles (e.g. fenbendazole). Since the introduction of benzimidazole drugs in the 1960s, they have been used broadly in a wide range of species for the control of parasitic helminths. These anthelmintic compounds inarguably have the most extensive therapeutic effect against diverse parasites species, excellent anti-parasitic efficacy, and low toxicity in treated animals. Fenbendazole is one of the safest drugs used in food animals in terms of food residues [2]. This is essential in the poultry industry where benzimidazoles are veterinary drugs extensively used for treatment and prevention of parasitic infestations. Levamisole is one of the imidazothiazole derivatives that is a potent and efficient anthelmintic for gastrointestinal nematode infestations in poultry, companion animals, and swine, and also against lungworms [2]. Following the encouraging findings in the poultry industry, levamisole has generally been used for the prevention and treatment of ascarid infections [3].

In recent years, anthelmintic resistance has been a prevalent problem in the control of gastrointestinal nematodes of animals in virtually every region of the world [4–6]. In Iran, studies assessing the field efficacy of anthelmintics and the detection of anthelmintic resistance have been performed since 2007[7]. Anthelmintic resistance (AR) in Iran has been described in gastrointestinal nematodes of sheep (benzimidazole and levamisole resistance) [7–12] and horses (benzimidazole resistance) [13]. Notably, some evidence on AR was not accessible because of the inevitable conditions (e.g. information not released for consideration by scientists, etc.). Moreover, these reports indicated a partial number of selected farms, and no additional supposition could be concluded for parasitic drug resistance at a local or national level. Until now, to our knowledge no study has tried to ascertain the field efficacy of anthelmintics in poultry. Consequently, the present study was conducted to assess the *in vivo* anthelmintic efficacy of fenbendazole and levamisole against the gastrointestinal helminths of chickens in northern Iran.

## Materials and methods

### Study area

**Fig. 1.**
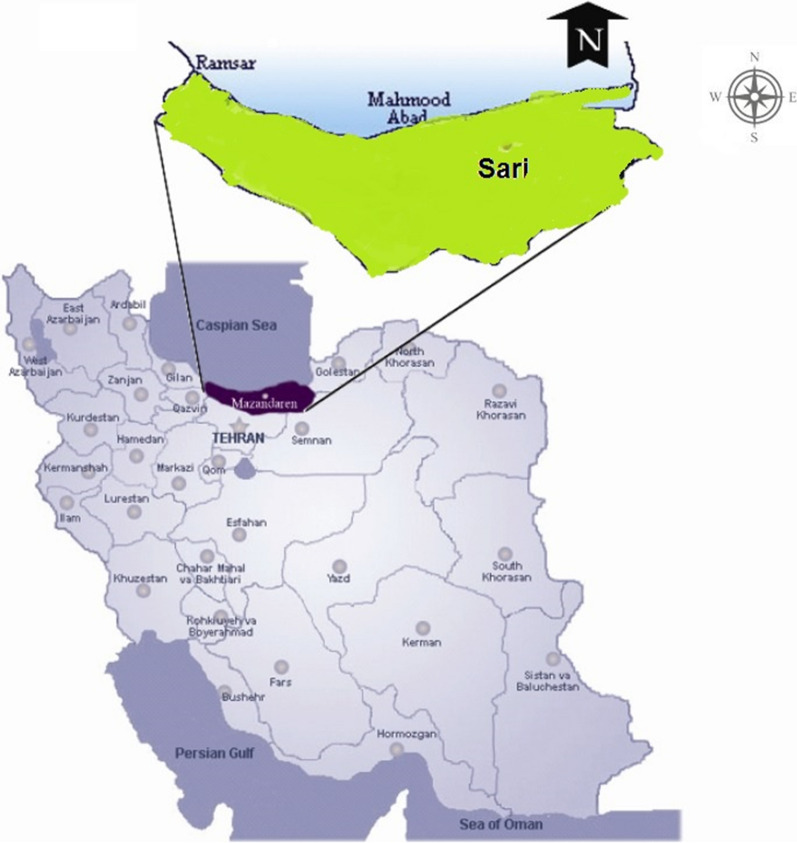
Map of Iran. The highlighted area is Sari City in Mazandaran Province

This study was conducted in naturally infected flocks of domestic chickens in Sari City in northern Iran (51°26′E, 35°41′N) from April 2017 to September 2018. Sari is the provincial capital of Mazandaran Province, located in the north of Iran between the northern slopes of the Alborz Mountains and the southern coast of the Caspian Sea (Fig. 1). Sari has a humid subtropical climate, with a Mediterranean climate influence. It lies approximately 32 m above sea level and has a mild climate characterized by 789.2 mm rainfall per annum. The mean annual temperature is 15 °C.

### Selection of flocks

Out of 45 domestic chicken flocks allocated to the study, 3 flocks were chosen to participate in fecal egg count reduction testing (FECRT) based on flock size. The following criteria were used: (i) the flock had 40–60 domestic chickens of different ages and sexes; (ii) anthelmintic drugs had not been used within the past 8 weeks; (iii) average fecal egg counts (FECs) were higher than or equivalent to 100 eggs per gram (EPG) of feces; (iv) there was a history of anthelmintic application on the flock in the last 3 years. When a farmer agreed to take part and met the first two criteria, fecal samples were collected from 20 randomly selected domestic chickens and tested for FEC. Over 45 flocks were tested to obtain 3 suitable flocks for the FECRT trial.

During the experiment, chickens were kept indoors and fed according to the norms of the field; water was administered *ad libitum*. Three to four days before the experiment was conducted, feces were examined using the McMaster method; then, domestic chickens were randomly assigned to experimental groups with a similar degree of infection to further determine the geometric mean number of eggs per gram of feces. 

### Fecal egg count reduction test (FECRT)

The FECRT was done for each flock, according to the World Association for the Advancement of Veterinary Parasitology (WAAVP) guidelines for the assessment of anthelmintic efficacy in poultry [14]. The 90 infected chickens were randomly distributed into three equal groups of 30 each. Each group was kept in a separate wire-floored cage (550 cm^2^/chicken). The chickens, naturally infected with helminths, were randomly divided into three experimental groups as follows with ten chickens in each, and one group was considered the control.

Group 1: 5 mg kg^−1^ body weight (BW) of fenbendazole for 3 consecutive days.

Group 2: 16 mg kg^−1^ BW of levamisole.

Group 3: placebo, water + DMSO (the control).

Drug doses were calculated based on a recent study on the efficacy of the fenbendazole and levamisole against internal parasites in chickens [15, 16]. To standardize the doses of fenbendazole and levamisole, all chickens were treated individually as the doses were given orally via syringe with a blunt needle.

### Fecal egg counts (FEC)

Fresh fecal samples were collected from a random sample of chickens (30% of each infected group). Selected chickens were marked, and only on the day of sampling was each chicken placed into an individual cage for defecation. Three grams of fecal samples was collected from the initially selected chickens on d 0 (for pre-treatment FEC) and d 14 post-treatment in zip-lock plastic bags and kept at 4 °C until processed for FEC using a modified McMaster technique [17]. A decrease in % FEC describes the percent of reduced FEC between the negative control and treated groups [17]. It was calculated on d 14 post-treatment according to the following formula:

$$\% {\text{ FEC reduction test }}\left( {{\text{FECRT}}} \right) \, = {1}00 \, \times \, \left( {{\text{FEC control}} - {\text{FEC treated}}} \right) \div {\text{FEC control}}$$

### Necropsy

For postmortem examination, chickens were killed and dissected on day 14 after dosing to obtain worms [18]. Immediately, the gastrointestinal tract of each chicken was removed, and the small intestine was separated. The contents of the intestine were washed into a sieve of 100 µm pore size and inspected for the presence of adult helminths. Subsequently, all visible worms were collected. Evaluation of anthelmintic efficacy was calculated according to the guidelines of the World Association for the Advancement of Veterinary Parasitology (WAAVP) for assessing the efficacy of anthelmintics in turkeys and chickens [14]. The efficacy of the treatments was evaluated by comparing FECs in the treated and control groups.

### Statistical analysis

Statistical analysis was performed with SPSS 19.0 software (SPSS Inc, Chicago, IL, USA) with *P* < 0.05 considered statistically significant. Repeated measures ANOVA was used to compare the differences between the experimental and control groups of animals.

The effectiveness of anthelmintic drugs was determined by the “control test” method according to the results of coproscopic examinations of animals with the calculation of the geometric mean value of the number of helminth eggs in samples before and 15 days after deworming of animals from the experimental and control groups. The independent samples *t*-test was used to compare the efficacy of two anthelmintic drugs.

### Interpretation of the FECRT results

Anthelmintic resistance status was evaluated according to the WAAVP guidelines on AR based on the percentage of fecal egg count reduction (%FECR) and the upper (UCL) and lower (LCL) 95% confidence limits [18]. Therefore, each anthelmintic was confirmed as (i) effective when the %FECR and UCL were both ≥ 95% and the LCL was ≥ 90%, (ii) suspected resistant when %FECR was < 95% or LCL was < 90%, and (iii) ineffective/resistant when both %FECR was < 95% and LCL was < 90%.

### Ethical statement

The blinded, randomized, and placebo-controlled study was performed according to the guidance for the experimental study of pharmacological substances. The use of domestic chickens in this study was approved by the Animal Ethics Committee (AEC no. 45403.3.1) of the Ferdowsi University of Mashhad.

## Results

Examination of three flocks of chickens from the control group showed that the animals were 95.0% infected with gastrointestinal nematodes (GINs) with an average geometric value of 361 eggs per gram of feces. Fenbendazole at a dose of 5 mg^−^kg BW for 3 days against GINs showed an efficacy of 83.7% (*P* ≥ 0.05), and levamisole at a dose of 16 mg^−^kg BW showed 71.8% efficacy (*P* ≥ 0.05) with geometric mean eggs per gram of feces of 100 and 199.6 for fenbendazole and levamisole, respectively. In general, both fenbendazole and levamisole showed low activity against GINs (Tables 1, 2).

**Table 1:** Data on farms included in the fecal egg count reduction test in domestic chickens in northern Iran from April 2017 to September 2018

Farm	Group	Group size	Group mean EPG pre	Group mean EPG post	Max individual EPG prec	Max individual EPG postc	Efficacy mean of drug
1	1(control)	5	215.8	212.6	625	606	–
	2(fbz)	5	251.8	59.2	583	134	79.38%
	3(lev)	5	398.8	213.8	602	525	43.81%
2	1(control)	7	141.7	139	337	332	–
	2(fbz)	7	137.6	22.9	263	85	85.18%
	3(lev)	7	326.7	137	628	38	89.81%
3	1(control)	5	84.4	89.4	149	147	–
	2(fbz)	5	178.8	29	294	81	86.68%
	3(lev)	5	120.4	15.2	218	36	81.82%

**Table 2:** Helminth egg species observed in domestic chickens in northern Iran from April 2017 to September 2018

Farm		1			2			3	
	cont	fbz	lev	cont	fbz	lev	cont	fbz	le
Species of helminth egg observed pre-treatment	*Cap* *Asc* *Trich* *Amid*	*Cap* *Asc* *Trich* *Amid* *Syn*	*Cap* *Asc* *Trich* *Amid* *Syn*	*Cap* *Asc* *Trich* *Syn*	*Cap* *Asc* *Trich* *Amid* *Syn*	*Cap* *Asc* *Trich* *Syng*	*Cap* *Asc* *Trich* *Amid*	*Cap* *Asc* *Trich* *Amid*	*Cap* *Asc* *Tric* *Ami*
Species of helminth egg observed post-treatment	*Cap* *Asc* *Trich* *Amid*	*Cap*	*Cap* *Trich*	*Cap* *Asc* *Trich* *Amid*	*Cap*	*Cap*	*Cap* *Asc* *Trich* *Amid*	*Cap* *Trich* *Amid*	*Cap* *Tric* *Ami*

cont: control, fbz: fenbendazole, lev: levamisole

The FECRT results of this study showed that there was no significant difference between the groups treated with fenbendazole and levamisole and the control group. Also, there was no significant difference between the EPG of the control group and the treatment groups at the beginning of treatment.

**Fig. 2.**
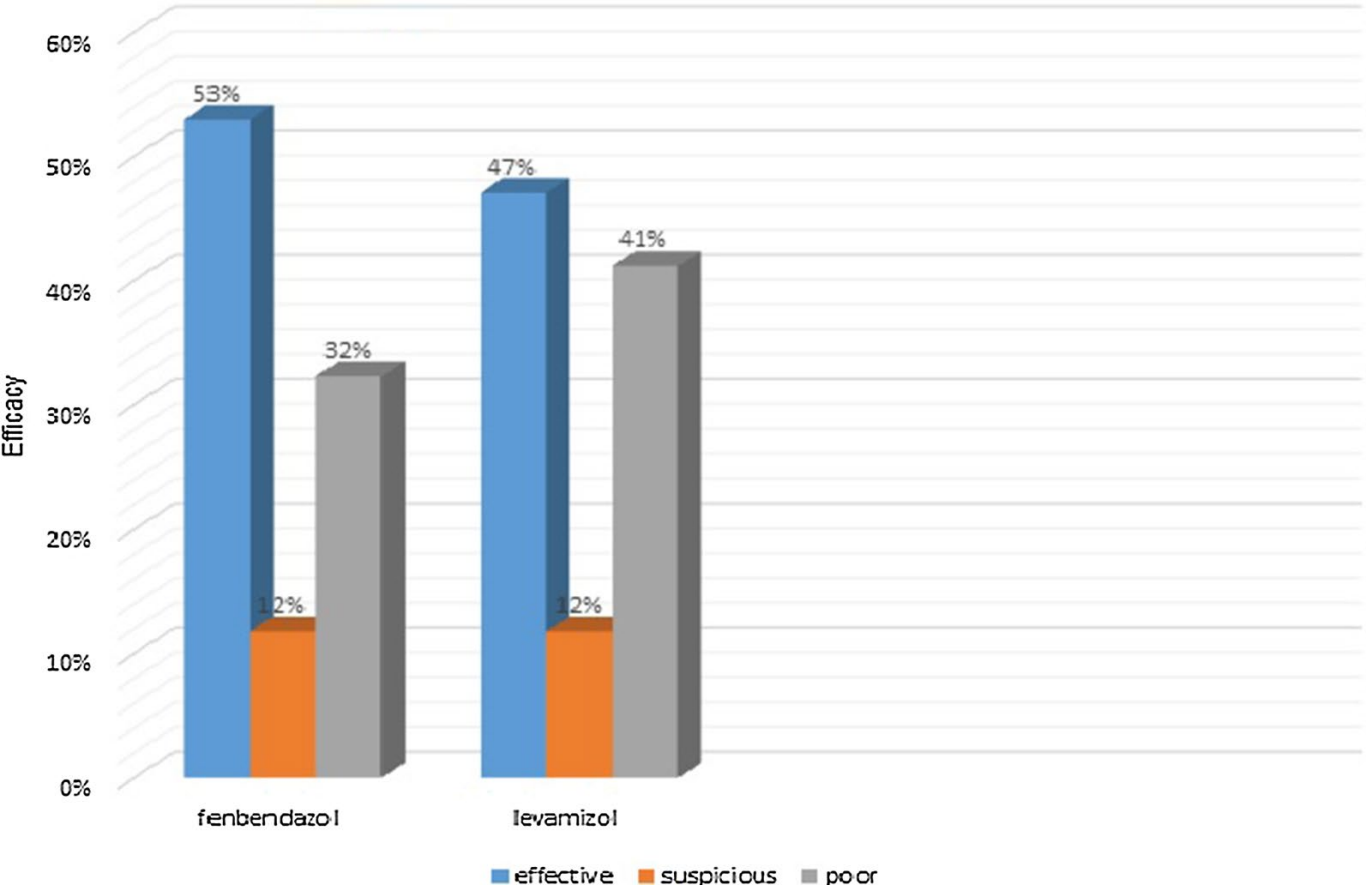
Proportion of farms with resistance, suspected resistance, and susceptibility of gastrointestinal nematodes of domestic chickens to two anthelmintics on 20 farms in Iran.

The FECRT results revealed that fenbendazole was more effective (83.7%) than levamisole (71.8%), but the difference was not significant (Fig. 2). The pre-treatment egg identification of GINs from all flocks of domestic chickens showed mixed GIN infections containing *Capillaria* spp., *Ascaridia galli*/*Heterakis* spp., *Syngamus trachea*, *Trichostrongylus spp.*, and *Amidostomom anseris* (Table 3).

**Table 3:** Necropsy results in farm 2 after treatment of the domestic chickens in northern Iran from April 2017 to September 2018

Group	Mean efficacy of drug	Helminth species observed post treatment in farm 2
Control	–	*Ascaridia galli* *Symgamus trachea* *Capillaria obsignata*
Fenbandazole	83.7%	*Capillaria annulata* *Capillaria obsignata*
Levamisole	71.8%	*Postharmostomum gallinarum* *Raillietina cestode*

*Capillaria* spp. were the most commonly resistant nematodes, followed by *Trichostrongylus* spp. and *A. anseris* (13%) whereas *A. galli/Heterakis *spp. and *S. trachea* were susceptible to the anthelmintics examined in this study.

Necropsy and worm burden of GINs in pre- and post-treatment groups of domestic chickens showed that fenbendazole more effectively removed all the GINs (i.e. *A. galli, Heterakis *spp. and *S. trachea*) prevalent in 20 domestic chicken flocks. Fenbendazole did not affect *Capillaria* spp. and *Trichostrongylus* spp., with a minimal effect on *A. anseris*. Likewise, levamisole seemed to have the highest efficacy against *S. trachea* while it was less effective against other GINs.

## Discussion

Despite the constant development in housing and management measures in poultry breeding, chickens kept on deep litter often must be treated for parasitic infections. The present study involves a field trial to investigate the efficacy of two anthelmintics, fenbendazole and levamisole, commonly used against GINs of domestic chickens. Our results indicated a reduced efficacy of fenbendazole and levamisole in three domestic Iranian chicken flocks. However, fenbendazole was a more effective anthelmintic than levamisole in the three Iranian domestic chicken flocks, but the difference was not significant. *Capillaria* spp. were the most generally resistant nematodes followed by *Trichostrongylus* spp. and *A. anseris*.

The AR of GINs against benzimidazole compounds in sheep [7] and horses [13] in Iran has previously been reported, but to our knowledge no previous data have been published on the field efficacy of benzimidazole compounds in poultry.

Various data are available on the therapeutic doses of fenbendazole and levamosole in chickens. However, the FECRT results of this study should be interpreted carefully, as we tested these two anthelmintics in domestic chickens based on the dose rates recommended in previous studies [15, 16]. Fenbendazole and levamisole are widely used broad-spectrum anthelmintic drugs in sheep, horses, and cattle in Iran. Anthelmintic resistance to BZs and levamisole has been reported for GINs of sheep from Iran [11]. Recently, Mohseni et al. [12] conducted a regional survey to evaluate the prevalence of AR in GINs of sheep in northeast Iran and found an average efficacy of 46% and 44% for albendazole and levamisole, respectively. Similarly, we found that fenbendazole was more effective (83.7%) than levamisole (71.8%). The reason for the reduction in sensitivity to levamisole in poultry flocks may be the frequent routine treatments, which impose strong selection pressure on worm populations and encourage the development of resistant strains. Because only three major chemical groups are currently available for the treatment of gastrointestinal and pulmonary nematodes, it is imperative for their usefulness to be conserved for as long as possible.

There are three main helminths that usually affect chickens—gapeworms, roundworms, and tapeworms. Fenbendazole and levamisole are generally used for the control of three species of helminth parasites of chickens including *A. galli*, *Heterakis gallinarum*, and *Capillaria* spp.; it has no adverse effect on the poultry egg-laying or hatching [2]. Of the species found in domestic chickens, *A. galli* heavily parasitizes free-ranging poultry [19, 20]; hence, control measures such as preventing infections or chemotherapy may improve weight gain and egg production. The results of our study revealed that fenbendazole and levamisole were effective in eliminating *A. galli*. It should be noted that evidence of fenbendazole resistance in *A. dissimilis*, a closely related ascarid of chickens, has been recently reported in turkeys with a history of heavy administration of frequent intervals of fenbendazole [21].

Moreover, *H. gallinarum* has been reported as another of the most frequent species of helminths in domestic chickens in Iran [19], and fenbendazole and levamisole are highly effective against it. Additionally, *Syngamus trachea*, which is a parasitic nematode that infects the tracheas of individual birds, is also common in young and domesticated chickens [22].

Concerning other nematodes, fenbendazole and levamisole do not affect *Capillaria* spp., which affects many species of wild birds and domesticated poultry, turkeys, and ducks [22]. The signs of *Capillaria* spp. infection can be challenging to identify because of the chronic nature of infection, and some non-specific signs include intermittent diarrhea, reduced appetite, ill-thrift, and weight loss [18]. *Capillaria* spp. in high numbers can be fatal to the chicken [22]. Given the ubiquity of *Capillaria* spp. and the possible economic impact, parasite control is an essential issue for bird health and productivity. Furthermore, *Trichostrongylus tenuis,* which is a gut nematode found in Iranian domestic chickens, does not have sensitivity to fenbendazole and levamisole. This endoparasite can cause poor condition and reproduction, leading to a condition called strongylosis or grouse disease [22].

The finding of resistance to fenbendazole and levamisole in *Capillaria* spp. and *Trichostrongylus* spp. may have far-reaching consequences for the poultry industry in terms of both animal welfare and economic impact. Given the results of our study along with the former finding of the suboptimal efficacy of fenbendazole in *A. dissimilis* [21], it seems that resistance can be expected in the helminth parasites of poultry. Subsequently, more extensively scaled surveys of resistance are needed to determine the prevalence of anthelmintic resistance in the poultry industry. Furthermore, studies addressing the production costs of drug-resistant *Capillaria* spp. are required to determine the financial impact on the industry.

## Data Availability

All data generated or analyzed during this study are included in this published article and its Additional files.
